# Microtubule-associated protein 1B (MAP1B)-deficient neurons show structural presynaptic deficiencies *in vitro* and altered presynaptic physiology

**DOI:** 10.1038/srep30069

**Published:** 2016-07-18

**Authors:** Felipe J. Bodaleo, Carolina Montenegro-Venegas, Daniel R. Henríquez, Felipe A. Court, Christian Gonzalez-Billault

**Affiliations:** 1 Laboratory of Cell and Neuronal Dynamics (CENEDYN), Department of Biology, Faculty of Sciences, Universidad de Chile, Santiago, Chile; 2 Center for Geroscience, Brain Health and Metabolism, Santiago, Chile; 3 Department of Physiology, Faculty of Biological Sciences, Pontificia Universidad Catolica de Chile, Santiago, Chile; 4 The Buck Institute for Research on Ageing, Novato, USA

## Abstract

Microtubule-associated protein 1B (MAP1B) is expressed predominantly during the early stages of development of the nervous system, where it regulates processes such as axonal guidance and elongation. Nevertheless, MAP1B expression in the brain persists in adult stages, where it participates in the regulation of the structure and physiology of dendritic spines in glutamatergic synapses. Moreover, MAP1B expression is also found in presynaptic synaptosomal preparations. In this work, we describe a presynaptic phenotype in mature neurons derived from MAP1B knockout (MAP1B KO) mice. Mature neurons express MAP1B, and its deficiency does not alter the expression levels of a subgroup of other synaptic proteins. MAP1B KO neurons display a decrease in the density of presynaptic and postsynaptic terminals, which involves a reduction in the density of synaptic contacts, and an increased proportion of orphan presynaptic terminals. Accordingly, MAP1B KO neurons present altered synaptic vesicle fusion events, as shown by FM4-64 release assay, and a decrease in the density of both synaptic vesicles and dense core vesicles at presynaptic terminals. Finally, an increased proportion of excitatory immature symmetrical synaptic contacts in MAP1B KO neurons was detected. Altogether these results suggest a novel role for MAP1B in presynaptic structure and physiology regulation *in vitro*.

The proper function of the central nervous system (CNS) relies on the establishment of a neuronal network that conveys information through synaptic contacts. Chemical synapses in the CNS are composed of presynaptic and postsynaptic terminals. The cytoskeleton of the presynaptic compartment is mainly composed of actin microfilaments^1^,^2^,^3^. However, ultrastructure analyses show that microtubules (MT) are also detected at presynaptic terminals^4^,^5^,^6^,^7^. These findings raise the possibility that cytoskeleton associated proteins with the ability to interact with the actin and MT cytoskeleton could regulate synaptic structure and physiology. Microtubule-associated protein 1B (MAP1B) contains both actin and MT binding domains^8^,^9^. MAP1B is synthesized as a polyprotein which is proteolytically cleaved to generate a light chain (LC1) and a heavy chain (HC)^10^, and is predominantly expressed in the nervous system^11^, where it has been described as the first microtubule-associated protein (MAP) to be expressed during early stages of development^12^,^13^. Most MAP1B functions are related to events occurring during development of the nervous system. Thus, MAP1B regulates axonal elongation and guidance, and neuronal migration^14^,^15^,^16^. Nonetheless, MAP1B expression persists during adult stages, specifically in brain areas showing high synaptic plasticity like the olfactory bulb, hippocampus and cerebellum^13^,^17^,^18^. Accordingly, MAP1B levels remain constant during synaptogenesis *in vitro*, where it has been proposed to be associated with the plasma membrane^19^. In recent years, numerous studies have also linked MAP1B functions with the regulation of the physiology and structure of postsynaptic glutamatergic dendritic spines. MAP1B is present in the dendrites of mature neurons^20^ and has been detected in high throughput experiments conducted to study the adult synaptic proteome^21^,^22^. MAP1B is found in postsynaptic densities of the cerebellar cortex^23^ and also co-distributes with a subset of F-actin positive dendritic spines present in mature neurons^24^. Moreover, MAP1B participates in the endocytosis of AMPA receptors in dendritic spines through its interaction with the postsynaptic protein GRIP1^25^, and during long-term depression in a mechanism involving Tiam and Rac1^26^,^27^. However, to date there is a lack of information about any putative role for MAP1B in presynaptic terminals of mammalian excitatory synapses. For instance, it has been described that MAP1B interacts with and regulates the degradation of the voltage dependent calcium channel Ca_v_2.2 which is located at presynaptic terminals^28^,^29^. Unlike vertebrate CNS synapses, the presynaptic terminals found at the *Drosophila* neuromuscular junctions (NMJ), are abundant in MT as the main cytoskeletal component, where they assemble as loops that promote the establishment and maintenance of synapses^30^,^31^. Interestingly, it has been broadly documented that *Futsch*, the MAP1B ortholog in *Drosophila*, is present at the NMJ only in presynaptic terminals, where it interacts with the MT cytoskeleton, promoting its stability and hence synaptic growth^30^,^32^. Also, *Futsch* regulates the density of presynaptic active zones and glutamate release, independent of its microtubule stabilizing properties^33^. With these studies in mind, the aim of this work was to analyze the presynaptic phenotype of mature hippocampal neurons derived from wild-type (WT) and MAP1B knockout (MAP1B KO) mice. We found that MAP1B-deficient neurons present an altered presynaptic phenotype and physiology, characterized by an immature structure and morphology. Our results support the idea that MAP1B-dependent synaptic regulation is not exclusively postsynaptic, but also affects the presynaptic terminals.

## Results

### MAP1B is expressed in the adult brain and in axons of mature neurons

**Figure 1:**
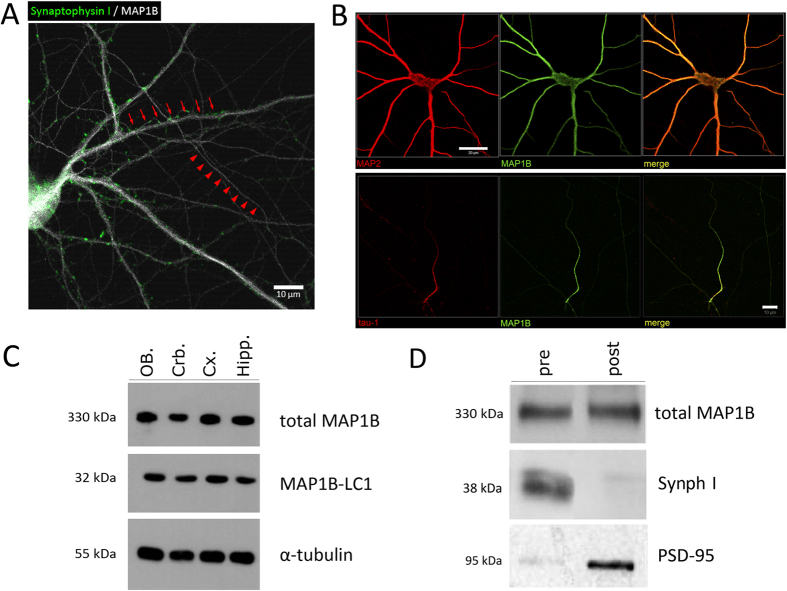
MAP1B is expressed in mature WT neurons and in adult brain. **(A)** Immunocytochemistry of a mature WT neuron stained against MAP1B and the presynaptic marker Synaptophysin I. Red arrows indicate dendrites innervated by presynaptic terminals, and red arrowheads indicate axons. Scale bar 10 μm. **(B)** MAP1B co-distributes with the somato-dendritic marker MAP2 (upper panel), and with the axonal marker tau-1 (lower panel). Scale bars 20 μm. **(C)** Immunoblot of mice adult brain tissue using antibodies against total MAP1B (upper panel) and MAP1B light chain (MAP1B-LC1, middle panel). 30 μg of proteins were loaded in each lane, and tubulin expression was used as a loading control. OB: olfactory bulb; Crb: cerebellum; Cx: cortex; Hipp: hippocampus. **(D)** MAP1B is present in both the presynaptic (Synaptophysin positive) and postsynaptic (PSD-95 positive) synaptosomal fractions derived from WT mice adult brain. For (**C**,**D**) gels for each antibody were run under same experimental conditions. Western blot raw data is presented in Supplementary information.

With the purpose of analyzing MAP1B expression and function, in the present work we cultured mature hippocampal neurons derived from E18.5 mice for immunocytochemistry (ICC) and electron microscopy analyses. For protein expression analyses by Western Blot, we used mature cortical neurons derived from E18.5 mice. Firstly, we determined that MAP1B is expressed in mature WT hippocampal neurons in thin prolongations that correspond to axons and in the thick processes corresponding to dendrites, which are innervated by presynaptic terminals positive for Synaptophysin I (Synph I) (Fig. 1A). Also, MAP1B co-distributes with specific somato-dendritic markers such as MAP2 or tau-1 in distal axons (Fig. 1B). By immunoblot analyses, we determined that both total MAP1B and its light chain LC1 are expressed in WT brain tissue at adult stages (2–3 month old animals) in cerebellum, cortex, hippocampus and olfactory bulbs (Fig. 1C). Also, MAP1B is detected in both presynaptic and postsynaptic synaptosomal fractions derived from WT mice adult brain (Fig. 1D). Uncropped immunoblots are available in Supplementary Information in the Western Blot raw data section. These results confirm previous reports of MAP1B in adult stages, and also highlight that in WT mature neurons, MAP1B is not only present in the somato-dendritic compartment but also in axons, where it could be involved in presynaptic terminal regulation.

### MAP1B deficiency does not changes the expression of synaptic proteins

**Figure 2:**
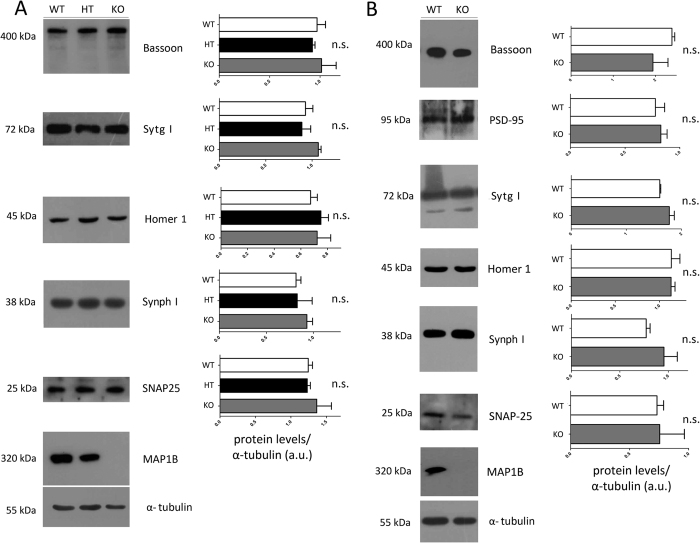
The expression level of a group of synaptic proteins is not altered in MAP1B KO neurons. Immunoblots against the presynaptic proteins Bassoon, Synaptotagmin I (Sytg I), Synaptophysin I (Synph I) and SNAP-25, and against the postsynaptic proteins Homer 1 and PSD-95. Expression of MAP1B in the different genotypes is shown, and tubulin was used as a loading control. 30 μg of protein were loaded in each lane. **(A)** Proteins derived from E18.5 embryonic brains of WT, MAP1B heterozygous and MAP1B KO mice. **(B)** Proteins from mature cortical neurons derived from WT and MAP1B KO mice. One–way ANOVA with Bonferroni post-test showed no significant differences between conditions, n = 3 for each condition (**A**). Unpaired Student’s t-test showed no significant differences between conditions, n = 3 for each condition (**B**). For (**A**,**B**) gels for each antibody were run under same experimental conditions and blot were processed in parallel. Western blot raw data is presented in Supplementary information.

In the next set of experiments, we studied if the absence of MAP1B could have an effect in synaptic maturation due to changes in the expression levels of presynaptic proteins. To answer this question, we prepared proteins extracts from WT and MAP1B KO neurons and performed immunoblots against the presynaptic proteins Bassoon, SNAP-25, Synaptotagmin I and Synaptophysin I, and the postsynaptic proteins Homer 1 and PSD-95. Since MAP1B KO mice die perinatally, we prepared protein extracts derived from E18.5 embryonic brain tissue and from a culture of mature cortical neurons. At the embryonic stage, we did not detect any statistically-significant variation in the expression levels of any of the synaptic proteins analyzed between WT, MAP1B heterozygous and MAP1B KO genotypes (Fig. 2A). Consistently, no differences in overall protein levels of synaptic proteins were observed between WT and MAP1B KO mature cortical neurons (Fig. 2B). Uncropped immunoblots are available in Supplementary Information in the Western Blot raw data section. These results show that MAP1B KO neurons do not present changes in the expression levels of the synaptic proteins analyzed by immunoblotting at different temporal stages.

### MAP1B KO mice display delayed SV fusion

**Figure 3:**
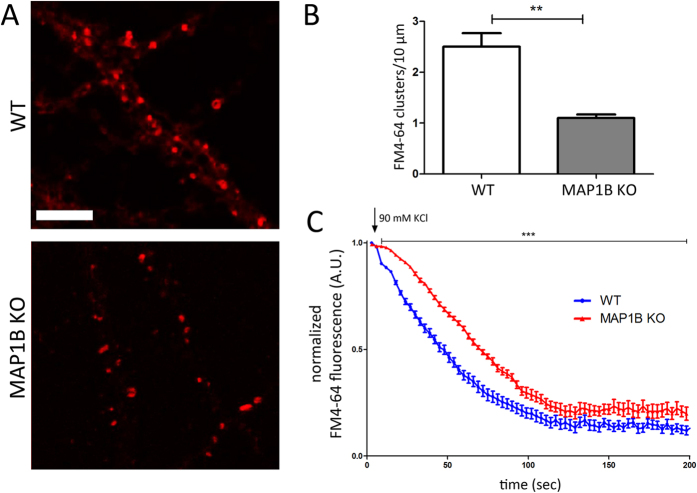
MAP1B KO neurons show delayed SV fusion events. **(A)** Representative images of mature neurons loaded with 10 μM of the fluorescent dye FM4-64 derived from WT (upper panel) and MAP1B KO (lower panel) mice. Scale bar 5 μm. **(B)** Quantification of the number of positive clusters for the FM4-64 dye (WT neurons, 2.5 ± 0.27 clusters/10 μm; MAP1B KO neurons, 1.1 ± 0.07 clusters/10 μm; unpaired Student’s t-test p < 0.01, 6091 μm analyzed for WT neurons and 6250 μm for MAP1B KO neurons. **(C)** FM4-64 normalized fluorescence over time graph after a depolarizing pulse of 90 mM KCl (signaled with arrow). The kinetics for WT and MAP1B KO neurons are shown with blue and red dots, respectively. All time points between 9 sec and 200 sec present statistically significant differences between genotypes, as determined by two-way ANOVA p < 0.001, n = 30 neurons per genotype.

The next step was to study whether MAP1B has an effect in synaptic transmission properties in mature neurons, by performing FM4-64 uptake. We prepared cultures of mature hippocampal neurons derived from WT or MAP1B KO genotypes. The mature WT and MAP1B KO neurons were loaded with the fluorescent dye FM4-64 during 1 minute of depolarization pulse and the kinetics of dye unloading induced by a second depolarization stimulus were analyzed^34^. This protocol allows the decrease in FM4-64 fluorescence intensity be quantified, as a direct measure of SV fusion in presynaptic terminals^35^. We first noticed that MAP1B KO neurons presented a 56% decrease in the number of FM4-64 positive clusters, which correspond to active presynaptic terminals (Fig. 3A,B). Moreover, when neurons were depolarized with 90 mM KCl, we observed a delay in the kinetics of SV fusion events in MAP1B KO neurons (Fig. 3C), raising the possibility that events prior to calcium influx like docking or priming of SV, could be affected in MAP1B KO presynaptic terminals. The fluorescence decay slopes (m) of both genotypes were slightly different between the 30 and 80 sec time intervals. The slope in MAP1B KO neurons is 8.86% steeper compared to WT neurons (WT, m = −0.0079 R^2^ = 0.987; MAP1B KO, m = −0.0086 R^2^ = 0.998). All time points between 9 sec and 200 sec present statistically significant differences between genotypes, and at the plateau stage (after 120 sec), MAP1B KO neurons retain 33.98% more average FM4-64 fluorescence intensity compared to WT neurons, raising the possibility that the pool of non-releasable SV is higher in MAP1B deficient neurons. Changes in the normalized fluorescence intensity over time were not related with FM4-64 probe photobleaching (Supplemental Fig. 1).

### MAP1B KO neurons display a reduced density of synaptic contacts

**Figure 4:**
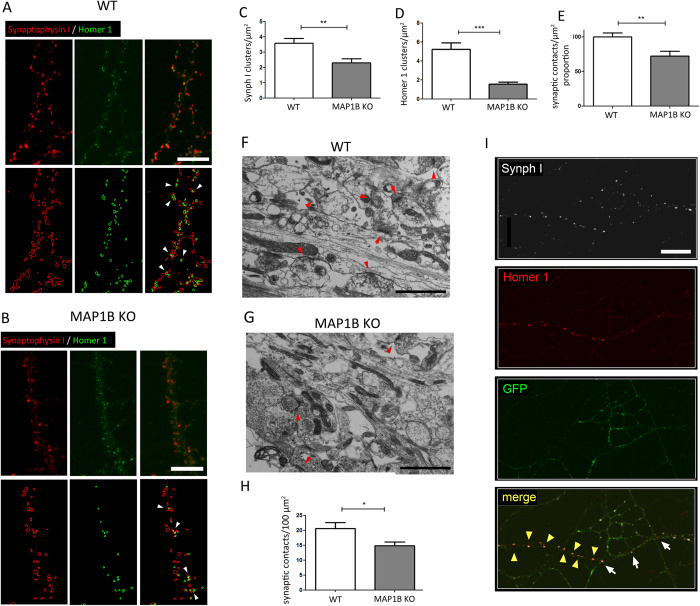
MAP1B KO neurons present a decrease in the density of synaptic contacts. Upper panels show an immunocytochemical analysis of mature neurons derived from **(A)** WT and **(B)** MAP1B KO mice against Synph I (red channel) and Homer 1 (green channel). Scale bars 5 μm. The lower panels show the respective ImageJ reconstructions, where red outlines represent Synph I-positive presynaptic terminals and green outlines represent Homer 1-positive postsynaptic terminals. White arrowheads show synaptic contacts as sites of colocalization of both fluorescent signals. **(C)** Quantification of Synph I clusters per unit area (WT neurons, 3.58 ± 0.32 Synph I clusters/μm^2^; MAP1B KO neurons, 2.30 ± 0.27 Synph I clusters/μm^2^; unpaired Student’s t-test p < 0.005). **(D)** Quantification of Homer 1 clusters per unit area (WT neurons, 5.23 ± 0.67 Homer 1 clusters/μm^2^; MAP1B KO neurons, 1.56 ± 0.21 Homer 1 clusters/μm^2^; unpaired Student’s t-test p < 0.0001). **(E)** Normalized proportion of synaptic contacts, given by the proportion of colocalizing pre and postsynaptic spots, as determined using the Puncta Analyzer plugin for ImageJ (WT neurons, 100.0% ± 5.45%; MAP1B KO neurons, 72.13% ± 7.04%; unpaired Student’s t-test p < 0.005). The total number of Synph I clusters analyzed was 5566 in WT neurons, and 4108 in MAP1B KO neurons, and the total number of Homer 1 clusters analyzed was 8195 in WT neurons, and 2821 in MAP1B KO neurons. For the WT condition, 16 reconstructions were generated, and for MAP1B KO, 17 reconstructions were analyzed. Representative micrographs for mature cultured **(F)** WT and **(G)** MAP1B KO hippocampal neurons. Red arrowheads show synaptic contacts. Scale bars 1 μm. **(H)** Quantification of total synaptic contact density determined by ultrastructure analysis (WT neurons, 20.62 ± 2.03 synapses/100 μm^2^; MAP1B KO neurons, 14.82 ± 1.29 synapses/100 μm^2^; unpaired Student’s t-test p < 0.05). **(I)** Mature WT control (GFP-negative) and MAP1B shRNA (GFP-positive) neurons, where the white channel corresponds to Synaptophysin I (Synph I), red to Homer 1 and green to GFP. This particular region was chosen because a single WT dendrite is innervated by both WT and MAP1B shRNA axons. White arrows indicate synapses between a presynaptic MAP1B shRNA axon (GFP-positive) and a WT dendrite. Yellow arrowheads indicate synapses between a WT presynaptic axon and a WT dendrite. Scale bar 10 μm.

**Figure 5:**
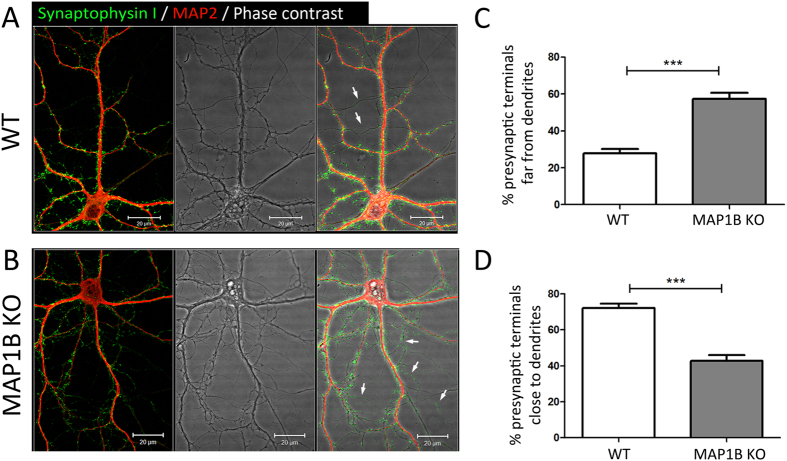
MAP1B KO neurons present an increased proportion of orphan presynaptic terminals. Representative immunocytochemical images of neurons derived from **(A)** WT and **(B)** MAP1B KO mice. The green channel corresponds to Synaptophysin I, and red to MAP2. Phase contrast images show that orphan presynaptic terminals are present along axonal compartments that are not in contact with dendrites (white arrows) in neurons of both genotypes, and do not correspond to unspecific signals or out-of-cell fluorescence. Scale bar 20 μm. Automated quantification of the proportion of presynaptic terminals: **(C)** far from dendrites (WT neurons, 27.81% ± 2,30%; MAP1B KO neurons, 57.32% ± 3.40% t-test p < 0,05) and **(D)** close to dendrites (WT neurons, 72.19% ± 2.30%; MAP1B KO neurons, 42.68% ± 3.40%, unpaired Student’s t-test p < 0.0001). Analyses were undertaken using 15 reconstructions for each genotype.

**Figure 6:**
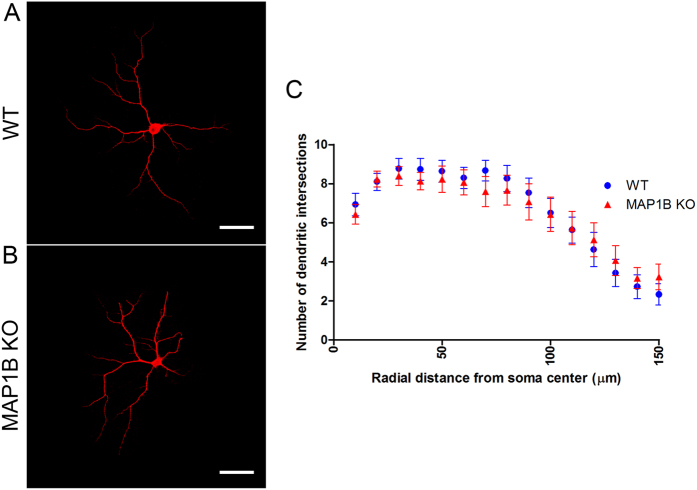
MAP1B KO neurons are unchanged in dendritic arbor complexity. Representative images of MAP2-stained WT **(A)** and MAP1B KO **(B)** mature hippocampal neurons. **(C)** Quantification of the intersection events between MAP2-positive dendrites and Sholl concentrical circumferences within 150 μm radial distance from the soma center. There are no differences between WT and MAP1B KO neurons at any of the radial distances analyzed. Two-way ANOVA p > 0.5 for each distance, n = 30 neurons for both genotypes.

By using ICC approaches, a synaptic contact can be defined as the region where the fluorescent signals of a presynaptic and a postsynaptic protein marker partially overlap^36^. Therefore, in order to determine synaptic density in our culture system, we performed ICC in mature WT and MAP1B KO hippocampal neurons. We used antibodies against Synaptophysin I (Synph I), a synaptic vesicle transmembrane protein that has been broadly-employed as presynaptic marker^37^,^38^, whilst the postsynaptic protein Homer 1, was used as a marker of the glutamatergic postsynaptic compartment^36^,^39^. We prepared z-stack reconstructions from randomly-selected cultured neurons immunostained against Synph I and Homer 1 for WT (Fig. 4A) and MAP1B KO neurons (Fig. 4B). We observed a reduction in the density of both total Synph I presynaptic and Homer 1 postsynaptic terminals in MAP1B KO neurons, by 35.8% (Fig. 4C), and 70.2% (Fig. 4D) compared to WT, respectively. We used a modified protocol based on previous reports^40^,^41^ that allows the quantification in a semi-automated manner of the proportion of colocalizing pre- and postsynaptic spots. The density of synaptic contacts given by the proportion of colocalizing spots was 27.9% lower in MAP1B KO neurons compared to WT neurons (Fig. 4A,B,E). Ultrastructure analyses through electron microscopy also showed a reduced density of total synaptic contacts in MAP1B KO neurons (Fig. 4F), from 20.62 ± 2.03 synapses/μm^2^ in WT neurons to 14.82 ± 1.29 synapses/μm^2^ in MAP1B KO neurons (Fig. 4G). This fall corresponds to a 28.1% decrease in synaptic contact density, a similar value to that found in ICC reconstructions (compare Fig. 4E,H). It is worth emphasizing that the total area of the substratum analyzed was similar between genotypes (736.90 μm^2^ for WT neurons, and 713.22 μm^2^ for MAP1B KO neurons). These results suggest that the capacity for establishing or maintaining synaptic contacts in mature neurons is decreased in neurons lacking MAP1B. It remains elusive whether the presynaptic defects observed in MAP1B KO neurons are due to a cell-autonomous presynaptic mechanism or a consequence of the altered postsynaptic structure and physiology in MAP1B KO neurons^24^. Therefore, we performed MAP1B knockdown (KD) in WT hippocampal neurons in order to obtain both WT and MAP1B shRNA axons contacting dendrites, in the same ICC sample. To differentiate control and KD neurons, MAP1B shRNA was co-nucleofected with GFP as a whole-cell marker. We observed that MAP1B KD axons (GFP-positive) establish fewer synaptic contacts than WT control axons (GFP-negative) as revealed by the Synph I-Homer 1 stain pattern (Fig. 4I). These results strongly suggest that at least a proportion of the presynaptic phenotype observed in MAP1B KO neurons relies on specific presynaptic axonal MAP1B roles; however, postsynaptic-dependent regulation cannot be discarded, as demonstrated previously^42^,^43^. MAP1B KD efficiency and other representative cases of the altered synapse establishment in MAP1B-deficient axons can be found in Supplemental Figs 2 and 3, respectively. Next, we studied the distribution of presynaptic terminals in mature hippocampal neurons by ICC, analyzing the localization of Synph I-positive presynaptic terminals relative to the dendritic marker MAP2. In both WT and MAP1B KO 21 DIV neurons, Synph I is expressed in a spotted fashion adjacent to MAP2-positive dendritic processes as expected, where 72.19% ± 2.30% of presynaptic terminals are in contact with a dendrite. However, irrespective of the genotype, there is a subset of presynaptic terminals which are not in contact with dendrites, a phenotype described-previously as orphan presynaptic terminals (OPT)^44^,^45^. In order to compare the prevalence of OPT in neurons of both genotypes, we performed z-stack reconstructions in randomly-selected samples. We set the following criteria to assess whether a Synph I spot corresponds to an OPT: each Synph I spot that is further than a given distance (2 μm) from a MAP2 positive dendrite is considered an OPT. It is important to mention that the presynaptic spots that do not contact dendrites are always located in axons, as observed in phase contrast images of neurons from both genotypes, thus ruling out the possibility that such Synph I clusters correspond to unspecific or out-of-cell staining. There is an increased proportion of OPT in MAP1B KO neurons (Fig. 5A,B) corresponding to an increment of 48.5% (Fig. 5C). As expected, there is a concomitant decrease in the proportion of presynaptic terminals in contact with dendrites (Fig. 5A,B) in MAP1B KO neurons (Fig. 5D). Although we seeded the same number of neurons for both genotypes, we cannot discard the possibility that axon branching could be increased in MAP1B KO neurons, as described before^46^. However, the increased proportion of OPT can be qualitatively observed in MAP1B KO neurons by phase contrast (Arrows, Fig. 5), independently of the axonal process density. To determine whether dendritic arbor complexity is altered in MAP1B KO neurons, we performed Sholl analyses. There are no differences in the Sholl index between genotypes, supporting the evidence that MAP1B KO neurons do not present changes in dendritic complexity when compared to WT (Fig. 6), as previously described^25^. These results show a decreased capacity to establish synapses in MAP1B-deficient neurons, a phenotype characterized by a reduction in presynaptic and postsynaptic terminals, as well as an increase in the proportion of OPTs.

### MAP1B KO neurons show decreased SV and DCV density at presynaptic terminals

**Figure 7:**
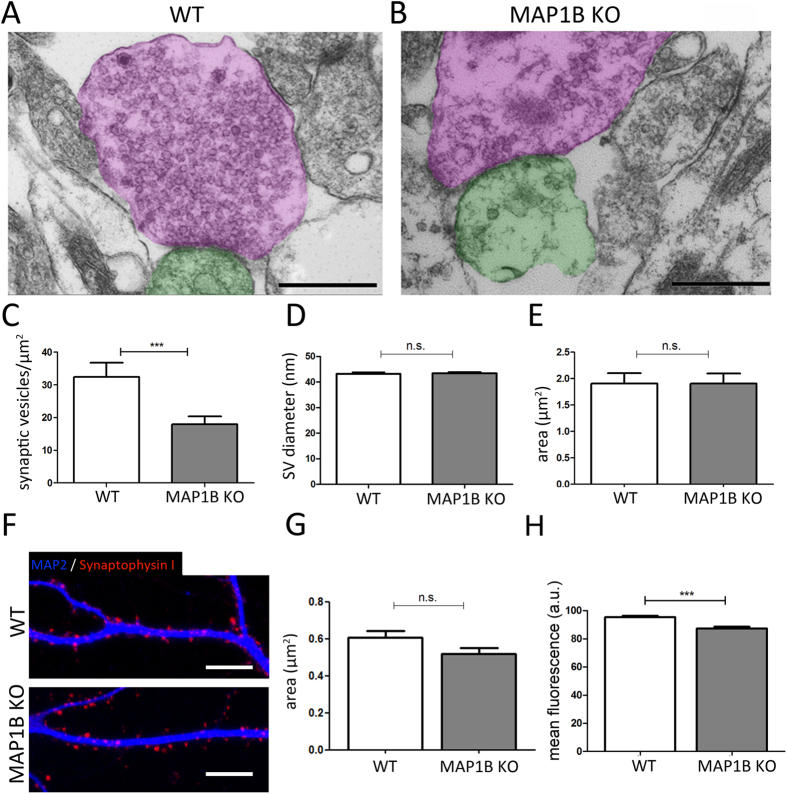
MAP1B KO neurons present a decrease in the synaptic vesicle density at presynaptic terminals, while their area is unaltered. Representative micrographs of synaptic contacts in (**A**) WT and (**B**) MAP1B KO mature neurons. Presynaptic terminals are pseudocolored in magenta, and postsynaptic terminals in green. Scale bars 500 nm. (**C**) Quantification of SV density in presynaptic terminals (WT neurons, 32.42 ± 4.38 vesicles/μm^2^; MAP1B KO neurons, 17.97 ± 2.40 vesicles/μm^2^; non-parametric Mann-Whitney test p = 0.0007). (**D**) Quantification of SV diameter (WT neurons, 43.23 ± 0.51 nm; MAP1B KO neurons, 43.39 ± 0.50 nm; unpaired Student’s t-test, not significant (n.s.)) (**E**) Quantification of presynaptic terminal surface area (WT neurons, 1.905 ± 0.20 μm^2^; MAP1B KO neurons, 1.903 ± 0.19 μm^2^; unpaired Student’s t-test n.s). (**F**) Representative immunocytochemical image of Synph I-positive presynaptic terminals in apposition to MAP2-positive dendrites in WT (upper panel) and MAP1B KO neurons (lower panel). Scale bar 10 μm. (**G**) Quantification of presynaptic terminal surface by ImageJ reconstruction (WT neurons, 0.605 ± 0.04 μm^2^; MAP1B KO neurons, 0.517 ± 0.03 μm^2^; unpaired Student’s t-test n.s). (**H**) Quantification of Synph I-positive presynaptic terminal mean fluorescence intensity by ImageJ reconstruction (WT neurons, 95.26 ± 1.07 a.u.; MAP1B KO neurons, 87.25 ± 1.39 a.u.; unpaired Student’s t-test p < 0.0001).

The changes observed in presynaptic terminals, suggest that MAP1B-deficiency could affect the content of synaptic vesicles. In order to evaluate such a possibility, we developed a high power magnification ultra-structural approach to analyze the density of synaptic vesicles (SV) and the area of presynaptic terminals in mature hippocampal neurons derived from WT (Fig. 7A) and MAP1B KO (Fig. 7B) mice. We observed that the density of SV located at presynaptic terminals is reduced by 44.6% in MAP1B KO compared to WT neurons (Fig. 7C); however, the SV diameter at presynaptic terminals is unaltered in neurons from both genotypes (Fig. 7D). Remarkably we did not observe any change in the surface area of presynaptic terminals^47^ between WT and MAP1B KO neurons (Fig. 7E). Similar results were obtained following z-stack reconstruction from the ICC approach, since there were no differences in the area of the Synph I presynaptic spots in WT and MAP1B KO neurons (Fig. 7F,G). However, following this protocol, we observed a decrease in the mean fluorescence intensity of Synphy I positive clusters (Fig. 7H), which is in direct relation with the reduced SV density observed through electron microscopy in MAP1B KO neurons.

**Figure 8:**
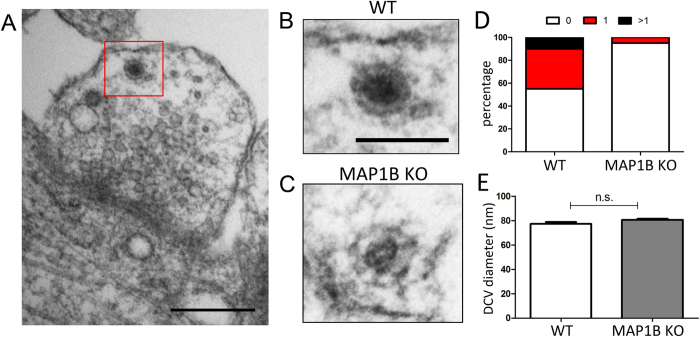
MAP1B KO neurons present a decrease in the dense core vesicle (DCV) density. **(A)** Representative micrograph of a DCV present in a presynaptic terminal of a mature WT hippocampal neuron. Scale bar 250 nm. Higher magnification of a representative DCV present in **(B)** WT and **(C)** MAP1B KO neuron. Scale bar 100 nm. **(D)** Proportion of presynaptic terminals with no DCV (white), with 1 DCV (red), and with more than 1 DCV (black) in mature neurons of both genotypes. **(E)** Quantification of DCV diameter (WT neurons, 77.47 ± 1.58 nm; MAP1B KO neurons, 80.75 ± 0.98 nm; unpaired Student’s t-test n.s).

Large dense core vesicles (DCV) (~100 nm diameter) contain neuropeptides and other co-transmitters, whereas smaller DCV (~80 nm diameter) harbor pre-assembled components of the presynaptic active zone^48^, which are also called Piccolo-Bassoon transport vesicles (PTV)^49^,^50^. With this in mind, we evaluated the presence of DCVs at presynaptic terminals (Fig. 8A). We found that DCVs, irrespective of the genotype, showed similar diameters (WT, 77.47 ± 1.58 nm; MAP1B KO, 80.75 ± 0.98 nm), which is consistent with the size of small DCV or PTV^48^ (Fig. 8B–D). While there were no variations in the size of such vesicle pools, the number of presynaptic terminals that contain at least one of these elements was severely-decreased in MAP1B KO derived neurons (Fig. 8E). Together, the reduction in the SV and DCV density pools observed in MAP1B KO neurons suggest that MAP1B deficiency leads to an abnormal presynaptic phenotype.

### Increased symmetrical synaptic contacts in MAP1B KO neurons

**Figure 9:**
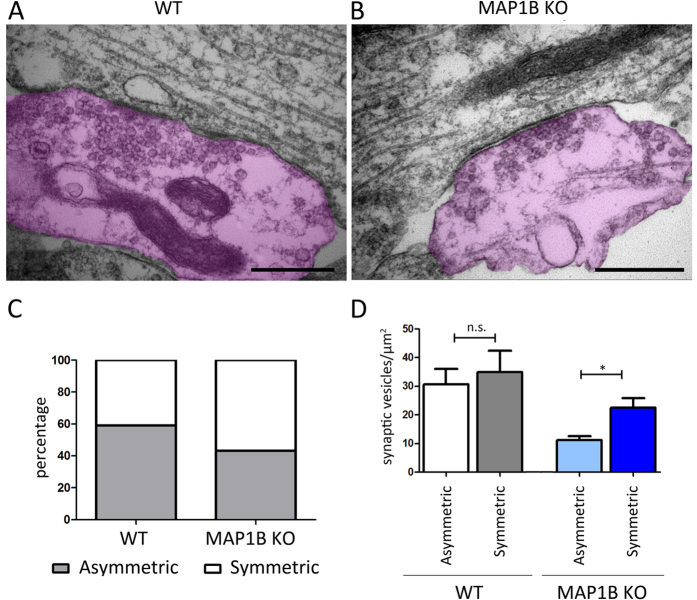
MAP1B KO neurons exhibit an increased proportion of symmetrical synaptic contacts. Representative micrograph of a symmetrical synapse in **(A)** WT and **(B)** MAP1B KO neurons, where the presynaptic terminals have been pseudocolored in magenta. Scale bar for (**A**) is 1000 nm, and for (**B**) 500 nm. **(C)** Quantification of the proportion synaptic contact types. For WT neurons, 59.15% are asymmetrical and 40.85% are symmetrical synapses. For MAP1B KO neurons, 43.30% are asymmetrical and 56.70% are symmetrical synapses. **(D)** Quantification of the SV density in each type of synapse. For WT neurons, asymmetrical synapses have 30.60 ± 5.49 SV/μm^2^, and symmetrical synapses have 34.91 ± 7.50 SV/μm^2^, non-parametric Mann-Whitney test n.s. For MAP1B KO neurons, asymmetrical synapses have 11.19 ± 1.37 SV/μm^2^, and symmetrical synapses have 22.50 ± 3.34 SV/μm^2^, non-parametric Mann-Whitney test p = 0.0122.

In addition to asymmetrical synaptic contacts that represent excitatory synapses^51^, we quantified the presence of symmetrical synaptic contacts that can be generated between an axonal presynaptic terminal and the dendritic shaft (Fig. 9A). We observed a 27.95% rise in the proportion of symmetrical synapses in MAP1B KO neurons compared to WT neurons (Fig. 9B). Moreover, we detected that while in WT neurons there are no variations in the SV density between symmetrical and asymmetrical synapses, MAP1B KO neurons exhibit a significant increase in SV density when symmetrical and asymmetrical synapses are compared (Fig. 9C). Alternatively, these symmetrical contacts may represent inhibitory synapses established in hippocampal neuron cultures^52^,^53^,^54^. Inhibitory synaptic contacts can be distinguished from axonal presynaptic and dendritic shafts contacts, based on the shape of their SV pool. It has been described that symmetrical synapses corresponding to inhibitory synaptic contacts display presynaptic terminals characterized by the presence of flattened and irregular SV morphology^53^,^54^,^55^. Therefore, in the final set of experiments we evaluated SV shape in symmetrical and asymmetrical synapses in both genotypes. We did not observe any significant changes either in the diameter or in the ellipticity of SV, irrespective of the type of synaptic contact and genotype analyzed (Supplemental Fig. 4). Therefore, these results suggest that MAP1B KO neurons exhibit an increased proportion of symmetrical synapses, which may correspond to immature excitatory synaptic contacts rather than inhibitory synapses.

## Discussion

In this work, we characterized the changes in synaptic contacts in long-term cultures of primary neurons derived from wild type and MAP1B-deficient neurons. While originally described as a protein involved in nervous system development, it has become apparent that MAP1B expression in adult brain is relevant for neuronal functions^21^,^22^,^56^. MAP1B expression remains elevated in regions of high brain plasticity such as the hippocampus, cortex and optic nerves^57^, and it has been previously shown that MAP1B regulates the morphology and physiology of postsynaptic dendritic spines in glutamatergic synapses^24^,^25^,^26^. In addition, MAP1B regulates Ca_v_2.2 channel expression in a mechanism involving its degradation by the ubiquitin proteasome system^29^. Therefore, it is interesting to integrate the role of MAP1B at both sides of the synapse. MAP1B KO neurons present a delay in the kinetics of FM4-64 fluorescence decay. This can be interpreted as an altered mechanism in stages prior to calcium influx in response to the arrival of the action potential, such as during the priming of SV^58^,^59^, or during the establishment of the calcium-independent interaction between the SNARE protein SNAP-25 and Synaptotagmin I^60^,^61^. These results open up the possibility that MAP1B in the presynaptic terminal exerts roles unrelated to its microtubule-stabilizing functions. One such novel function could be to regulate protein degradation at the presynaptic terminal^29^. Of note, paired pulse facilitation and post-tetanic potentiation experiments performed in acute hippocampal slices derived from heterozygous MAP1B animals showed no differences in the presynaptic function compared to a WT condition^26^. These results suggest that the aberrant presynaptic phenotype that we observed in mature neurons is detected only in the context of a complete deficiency of MAP1B. Such differential responses to MAP1B doses at pre- and post-synaptic terminals could be in fact due to local regulation of the protein levels dependent on selective expression of microRNAs miR-9 and miR-146a-5p^46^,^62^. Moreover, null models for the MAP1B homologous protein MAP1A exhibit defects in synaptic plasticity in hippocampal neurons and neurodegeneration at adult stages^63^,^64^, suggesting that microtubule-associated proteins regulate synaptic function.

Our long-term culture setup considers a homogenous population of neurons, in which MAP1B KO neurons display fewer synaptic contacts, and their presynaptic terminals present a decrease in SV and DCV density and a delay in SV fusion events. Moreover, considering that orphan presynaptic (OPT) terminals are suppressed during development and that their stabilization seems to be fundamental in the development of synapses^44^,^45^, such an increase in the proportion of OPT observed in MAP1B KO suggest that in the absence of MAP1B, synaptic maturation could be blocked. It is likely that the observed DCV component corresponds to Piccolo-Bassoon transport vesicles (PTV), which are involved in the transport of presynaptic components from the soma to the distal axon during synaptogenesis^48^,^50^. Thus, such reductions are in direct relation with the decreased capacity to establish and maintain synaptic contacts.

The establishment and maturation of a presynaptic terminal, as well as its physiological properties, are regulated by homeostatic mechanisms that partially depend on local postsynaptic dendritic activity^42^,^43^. Specifically, abnormal post-synaptic physiology triggers presynaptic defects characterized by a decreased number of presynaptic terminals contacting a target neuron, and smaller presynaptic vesicle pools^65^. For example, during stages prior to synaptogenesis, N-cadherin defective post-synaptic neurons receive fewer active presynaptic inputs, and the presynaptic terminals are smaller than in control conditions^66^,^67^. Our results strongly suggest that a proportion of the presynaptic phenotype observed in MAP1B KO neurons is caused by an altered presynaptic function, since MAP1B-deficient axons establish fewer synapses when contacting WT dendrites. However, it is important to mention that these observations do not rule out the possibility that the abnormal presynaptic phenotype observed in MAP1B KO neurons could partially depend on post-synaptic retrograde regulation, considering that MAP1B regulates the physiology and structure of dendritic spines in mature neurons.

Several synaptic maturation hypotheses propose that axonal-shaft synapses could correspond to immature glutamatergic synaptic contacts, and that these symmetrical contacts will eventually give rise to mature axo-spinal synapses^68^,^69^,^70^. In our WT neuron cultures, about 40% of synapses were asymmetrical, a proportion that is coherent with the described prevalence of symmetrical immature synapses *in vitro* attributed to the constant and non-pattern dependent process of synapse establishment, maintenance and elimination that occurs in culture^71^. Interestingly, in MAP1B KO neurons we observed an increased proportion of symmetrical synapses that could correspond to immature synaptic contacts, revealing a diminished capacity to consolidate and mature a synaptic interaction. It is important to mention that in brain tissue, the inhibitory synaptic contacts are also symmetrical, and possess pleomorphic and flattened SV^53^,^54^,^55^. Considering that we did not observe changes in the diameter and ellipticity of the SV between the types of synapses and genotypes, added to the fact that embryonic hippocampal neuron cultures have few inhibitory neurons^52^, the findings support the idea that immature symmetrical synapses are excitatory rather than inhibitory.

In conclusion, we present evidence showing that MAP1B deficiency leads to abnormal presynaptic terminal structure and physiology, and that a proportion of this phenotype is a consequence of an altered presynaptic mechanism in MAP1B-deficient axons. It remains to be elucidated whether such phenotypes are mostly dependent of MAP1B functions such as microtubule-stabilization or rather depends on novel MAP1B-specific functions.

## Materials and Methods

### Antibodies

The following primary antibodies were used in this study: mouse anti-α-tubulin (clone DM1A, T6199; Sigma-Aldrich), mouse anti-Bassoon (141021; Synaptic Systems), rabbit anti-Homer 1 (160021; Synaptic Systems), goat anti-MAP1B (N19; Santa Cruz Biotechnology), rabbit anti-MAP1B-LC1 (H130; Santa Cruz Biotechnology), rabbit anti-MAP2 (AB5622, Millipore), rabbit anti-PSD-95 (APZ-009, Alomone), rabbit anti-SNAP-25 (111002, Synaptic Systems), mouse anti-Synaptophysin I (101011; Synaptic Systems), mouse anti-Synaptotagmin I (105011; Synaptic Systems), and mouse anti-tau-1 (MAB3420, Millipore). Secondary antibodies for immunoblots were HRP-conjugated anti-mouse and anti-rabbit IgG (Jackson Laboratories), and HRP-conjugated anti-goat IgG (Santa Cruz Biotechnology). Secondary antibodies for immunocytochemistry were anti-mouse, anti-rabbit and anti-goat conjugated to Alexa-Fluor 488, 543 or 633 (Thermo Fischer).

### Immunoblot

Protein extracts from 13 DIV cortical neurons and from E18.5 embryonic brain were obtained using radioimmunoprecipitation assay (RIPA) buffer. Denaturated samples were separated by molecular weight by SDS-polyacrylamide gel electrophoresis (PAGE), and transferred to nitrocellulose membranes, which were blocked with BSA 5% in TBS, and incubated with primary antibodies diluted in BSA 1% in TBS overnight at 4 °C. HRP-coupled secondary antibodies were then incubated and immunoreactive bands were visualized using enhanced chemiluminiscent substrate (ECL, Thermo Fischer).

### Primary cultures

For co-cultures, E18.5 hippocampal neurons and neonatal astrocytes were obtained as previously described^72^. All experiments were approved by the Bioethical Committee of the Faculty of Sciences, University of Chile, in accordance to the ethical rules of the Biosafety Policy Manual of the National Council for Scientific and Technological Development (FONDECYT). Briefly, to obtain astrocytes, the cortex was dissected from 2–3 neonatal (P0-P2) Sprague-Dawley rats and treated with 0.25% trypsin and 0.1 g/ml DNAse I (Roche) in HBSS (Gibco) for 30 minutes at 37 °C. The tissue was dissociated and the cells seeded over a multi-well flask. Astrocytes were maintained in MEM medium (Gibco) with 10% house serum (HS), 0.6% glucose and 1% penicillin-streptomycin (Plating medium), and rinsed every 2 days with PBS to avoid the proliferation of microglia, until cells reached a confluence of about 80%. The day after the neuronal culture, astrocyte medium was removed and replaced with Neurobasal medium (Gibco) containing 2% B27 (Gibco), 1% Glutamax (Gibco), 10 mM sodium pyruvate (Gibco) and 1% penicillin-streptomycin (Neuronal Maintenance medium). To obtain E18.5 hippocampal neurons, hippocampi of WT and MAP1B KO embryos (*Mus musculus*, NMRI) were dissected and treated with 0.25% trypsin for 30 minutes at 37 °C. Neurons were seeded in Plating medium for 90 minutes at a low density (10,000 cells/cm^2^) over coverslips previously-treated with poly-L-lysine 1 mg/ml (Sigma-Aldrich) and dotted with wax. Subsequently, the coverslips were transferred to the previously-cultured astrocytes in Neuronal Maintenance medium, in such a manner that the neurons were orientated towards the bottom of the well, with no physical contact between neurons and astrocytes. At 3 DIV, neurons were treated with 5 μM AraC (Cytosine β-D-arabinofuranoside, Sigma-Aldrich) to inhibit proliferation of non-neuronal cells. For E18.5 cortical neuron culture, both cortical hemispheres of WT and MAP1B KO embryos were dissected and treated with 0.25% trypsin and 0.1 g/ml DNAse I for 30 minutes at 37 °C. Neurons were seeded in Plating medium for 60 minutes at a high density (50,000 cells/cm^2^) over plates treated with poly-L-lysine 1 mg/ml. The medium was then replaced with Neuronal Maintenance medium with N2 supplement (Gibco). All cultures were maintained at 37 °C with 5% CO_2_.

### MAP1B knockdown assay

For MAP1B knockdown in E18.5 hippocampal neurons, cells were nucleofected prior to plating according to instructions provided with the Amaxa Basic Nucleofector Kit for Primary Neurons (Lonza). Briefly, 1.5 × 10^6^ neurons were co-nucleofected with 1 μg pmax-GFP (Lonza) and 4 μg MAP1B shRNA (Broad Institute) using program O-005, as suggested by the manufacturers. After nucleofection, neurons were seeded at a density of 50,000 cells/cm^2^. For MAP1B knockdown in cell lines, full-confluent p60 plates of neuroblastoma N2a cells were transfected using 8 μl Lipofectamine 2000 (Invitrogen) and 4 μg MAP1B shRNA, and maintained in Dulbecco’s Modified Eagle’s Medium (Gibco) with 5% Fetal Bovine Serum (Gibco) for two days until cell lysis.

### Immunocytochemistry

Neurons were fixed with 4% PFA/4% sucrose diluted in PBS, for 30 minutes at 37 °C, and then washed three times with PBS and permeabilized with 0.2% Triton X-100 in PBS for 5 minutes. Neurons were blocked with 5% BSA in PBS for 90 minutes at room temperature. Primary antibodies were diluted in 1% BSA in PBS and incubated overnight at 4 °C in a humid chamber. Samples were washed three times with PBS, and the fluorophore coupled secondary antibodies were diluted in 1% BSA in PBS and incubated for 60 minutes at room temperature. After 3 washes with PBS, coverslips were mounted in FluorSave reagent (Millipore). Samples were analyzed on a Zeiss LSM710 confocal microscope.

### Synaptosomes purification

The protocol was performed as previously described with modifications^73^. Briefly, adult mice brain were homogenized in 0.32 Sucrose, 1 mM MgCl_2_, 0.5 mM CaCl_2_, 1 mM NaHCO_3_, 1 mM okadoic acid and 1 mM DTT and centrifuged at 1.440 g for 10 min. Pellet obtained was re-centrifuged and both supernatants were mixed and centrifuged at 13.800 g for 10 min. Resulting supernatant was centrifuged at 100.000 g for 1 hour. The resulting pellet corresponded to the synaptosomal fraction, which was resuspended in 0.32 Sucrose, 1 mM NaHCO_3_, 1 mM EGTA diluted in cold 0.1 mM CaCl_2_. Next was added 40 mM Tris, 2% Triton X-100 pH = 6.0, and samples were centrifuged at 40.000g for 30 min. Pellet was resuspended in 500 ml of 20 mM Tris, 2% Triton X-100 pH = 8.0 and centrifuged at 33.500 rpm for 30 min. The pellet obtained corresponded to post-synaptic densities and supernatant to presynaptic proteins. All procedure was done at 4 °C.

### ImageJ reconstruction analyses

Images were acquired in z-stack format with a Plan-Apochromat 63×/1.4 objective with a resolution of 1024 × 1024 pixels. All images were taken within 100 μm of the neuronal soma. Laser intensity, detector gain and scanning speed were unaltered for every image set analyzed. Maximum intensity projection of z-stacks was performed, and binary masks were generated for the fluorescent signal corresponding to Synaptophysin I, Homer 1 and MAP2 as appropriate, using the Adjust Threshold tool. For the synaptic contact analysis, the colocalization of pre and postsynaptic spots was performed with the Puncta Analyzer plugin, as described previously^40^,^41^. In this way, the total number of spots and the colocalization proportion were automatically obtained. For orphan presynaptic terminal (OPT) quantification, the MAP2 and Synaptophysin I binary masks were selected with the Analyze Particles tool. MAP2 masks were expanded with the Maximum filter in order to determine the area of contact with presynaptic terminals. Images were then merged with the Image Calculator > Multiply tool, and the proportion of presynaptic terminals close to or far from dendrites was automatically obtained. All statistical analysis was undertaken with Prism Graphpad software.

### Sholl Analyses

Mature hippocampal neurons of both genotypes were stained with the somato-dendritic marker MAP2 in order to analyze dendritic arbor complexity. The Sholl technique was performed using the open-source Sholl Analysis for ImageJ/Fiji plugin^74^. Briefly, binary masks for the MAP2 channel were generated for each isolated neuron analyzed, which were then subjected to Sholl analysis. Concentrical circumferences started from the center of the neuronal soma were generated, where the starting radius was 10 μm, the radius step size was 10 μm, and the final radius was 150 μm. The Sholl index was defined as the number of dendritic branches that cross the concentrical circumferences at each radius.

### Electron Microscopy

The protocol was performed as previously described^75^. Briefly, E18.5 hippocampal neurons were seeded over acetate cellulose coverslips (Electron Microscopy Science) at a density of 50,000 cells/cm^2^, and maintained in co-culture with astrocytes until 21 DIV. Neurons were fixed with 2.5% glutaraldehyde, 0.01% picric acid, 0.1 M cacodylate buffer, pH 7.4. Then neurons were incubated with 1% osmium tetroxide and 2% uranyl acetate, dehydrated with ethanol and propylene oxide, and finally infiltrated with Epon (Ted Pella Inc.). Ultrathin sections were contrasted with 1% uranyl acetate and lead citrate. Grids were analyzed in a Phillips Tecnai 12 electron microscope. Negative films were scanned and analyzed with ImageJ software.

### FM4-64 dye release assay

Mature hippocampal neurons were washed with a modified Tyrode solution (2 mM CaCl_2_; 2 mM MgCl_2_, 119 mM NaCl, 2.5 mM KCl, 30% glucose and 25 mM HEPES pH 7.4) with 50 μM APV and 10 μM CNQX. Subsequently, neurons were loaded with 10 μM FM4-64 dye (Molecular Probes) for 30 seconds in darkness, with a depolarization pulse of 30 mM KCl for 1 minute (first stimulus). Unspecific signals were removed with serial washes of Tyrode solution for 10 minutes. Coverslips were placed in a temperature-regulated chamber of a Zeiss LSM510 confocal microscope. A base line was acquired for 1 minute, and then neurons were depolarized with 90 mM KCl (second stimulus), which produced the decay in the fluorescence signal corresponding to FM4-64 dye. Images were taken every 2 seconds for 300 seconds. All recordings were done at 25 °C. Images were acquired with a 63 × 1.3 N.A. objective at a resolution of 512 × 512 pixels. The fluorescent dye was excited with a 543 nm laser and detected over 685 nm.

### Statistical analyses

All data represent means ± SEM of at least three independent experiments. Comparisons between two groups were made using unpaired Student’s t-tests when data presented a Gaussian distribution, and non-parametric Mann-Whitney tests when data presented non-Gaussian distribution. Comparisons between more than two groups were made using one-way ANOVA followed by Bonferroni’s post-test. Comparisons of grouped data sets were made using two-way ANOVA followed by Bonferroni’s post-test. Normality of data was determined using the Shapiro-Wilk normality test. A value of p < 0.05 was considered significant. Slope measurements were performed by linear regression.

## Additional Information

**How to cite this article**: Bodaleo, F. J. *et al*. Microtubule-associated protein 1B (MAP1B)-deficient neurons show structural presynaptic deficiencies ^in vitro^ and altered presynaptic physiology. *Sci. Rep.* **6**, 30069; doi: 10.1038/srep30069 (2016).

## Supplementary Material

Supplementary Information
