# Corrigendum: Microtubule-associated protein 1B (MAP1B)-deficient neurons show structural presynaptic deficiencies *in vitro* and altered presynaptic physiology

**DOI:** 10.1038/srep32275

**Published:** 2016-09-02

**Authors:** Felipe J. Bodaleo, Carolina Montenegro-Venegas, Daniel R. Henríquez, Felipe A. Court, Christian Gonzalez-Billault

Scientific Reports 6: Article number: 3006910.1038/srep30069; published online: 07 18 2016; updated: 09 02 2016

In this Article, an additional affiliation for Felipe A. Court has been omitted. The correct affiliation is listed below:

Center for Integrative Biology, Universidad Mayor, Chile.

