# Supplementary material for: Microtubule-associated protein 1B (MAP1B)-deficient neurons show structural presynaptic deficiencies in vitro and altered presynaptic physiology
Source: Sci Rep. 2016 Jul 18;6:30069. doi: 10.1038/srep30069 (PMC4948024; doi:10.1038/srep30069)
Supplement: Supplementary Information [file srep30069-s1.pdf]

Microtubule-associated protein 1B (MAP1B)-deficient neurons show structural presynaptic deficiencies *in vitro* and altered presynaptic physiology

Felipe J. Bodaleo[1,2], Carolina Montenegro-Venegas[1,5], Daniel R. Henríquez[1,2], Felipe A. Court[2,4] and Christian Gonzalez-Billault[1,2,3].

[1]Laboratory of Cell and Neuronal Dynamics (CENEDYN), Department of Biology, Faculty of Sciences, Universidad de Chile, Santiago, Chile

[2]Center for Geroscience, Brain Health and Metabolism, Santiago, Chile

[3]The Buck Institute for Research on Ageing, Novato, USA

[4]Department of Physiology, Faculty of Biological Sciences, Pontificia Universidad Catolica de Chile, Santiago, Chile

[5]Present address: Department of Neurochemistry/Molecular Biology, Leibniz Institute for Neurobiology, 39118 Magdeburg, Germany

Supplemental Figure 1

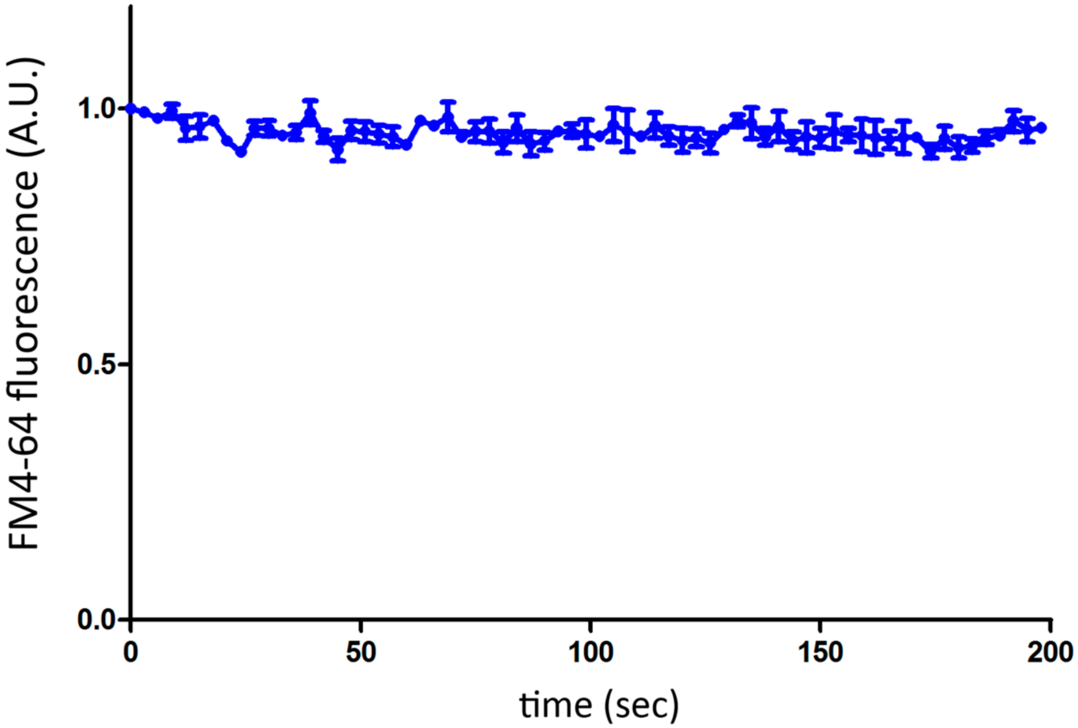

Supplemental Figure 2

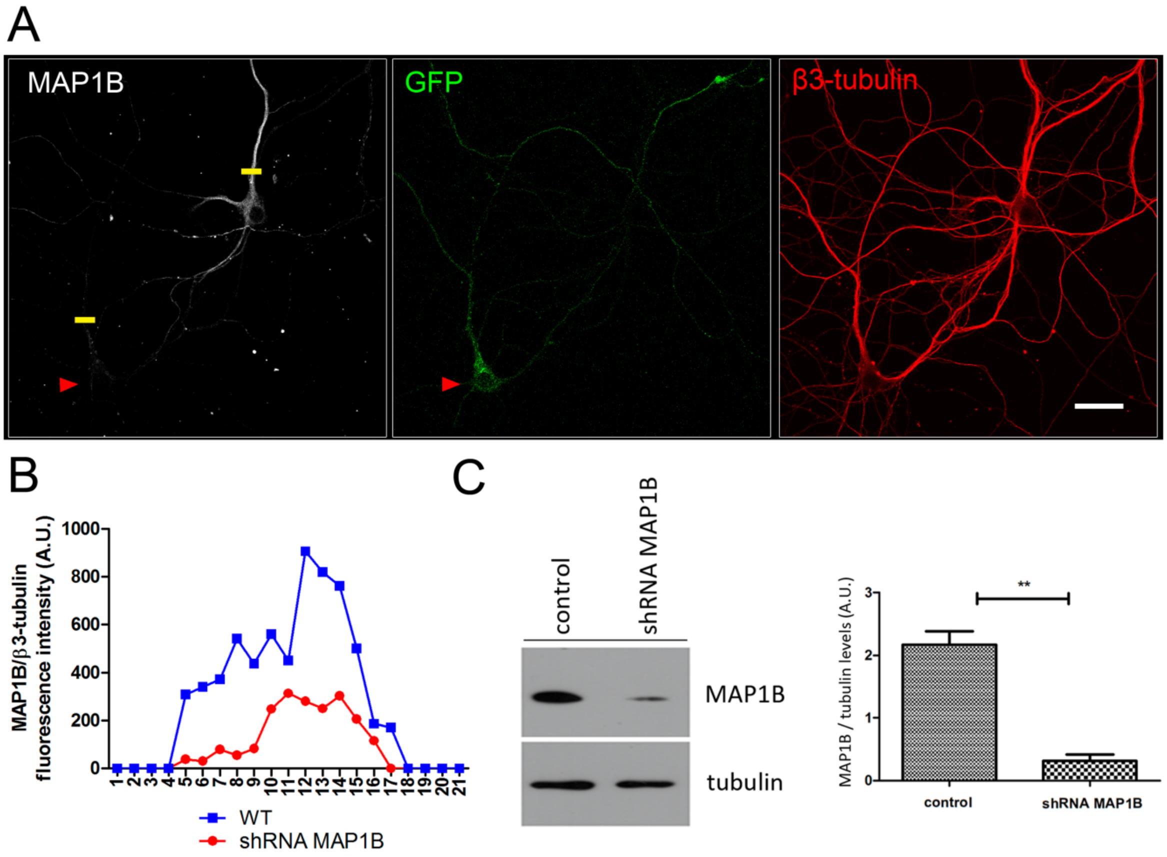

Supplemental Figure 3

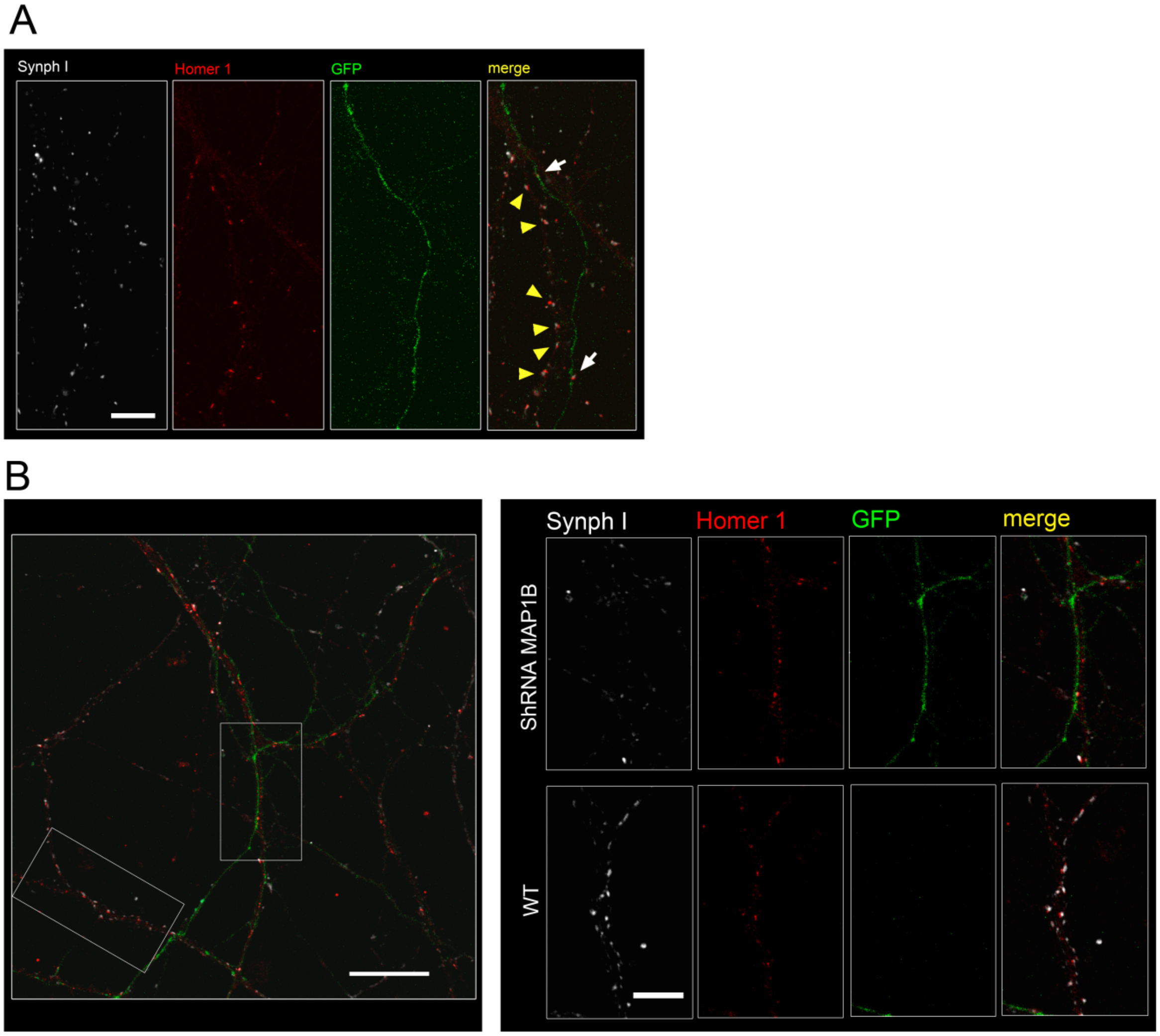

Supplemental Figure 4

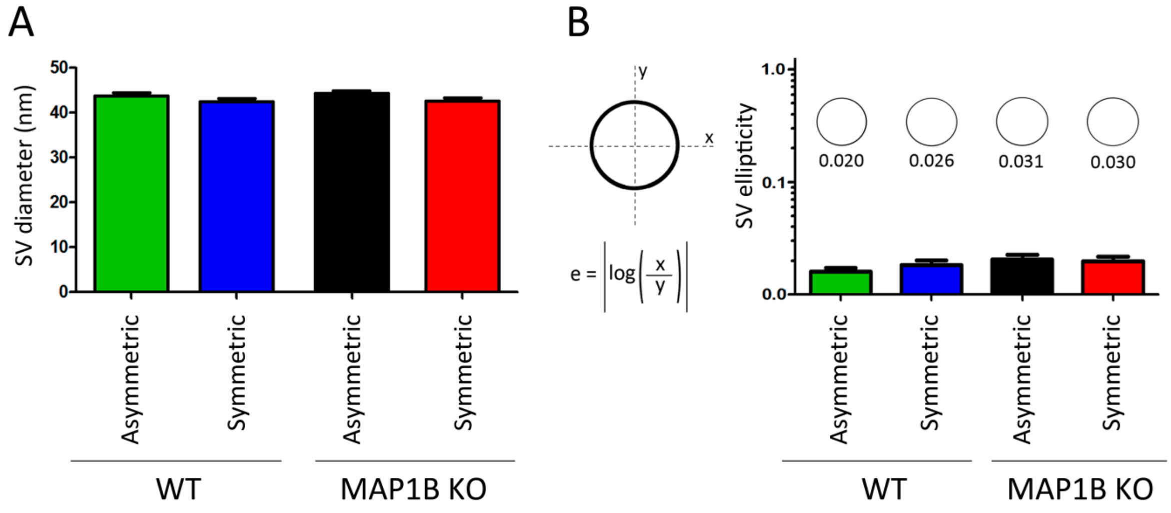

Western Blot raw data

Figure 1 C

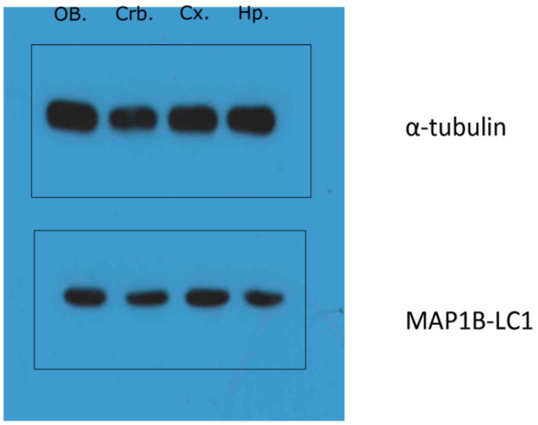

Figure 2 A

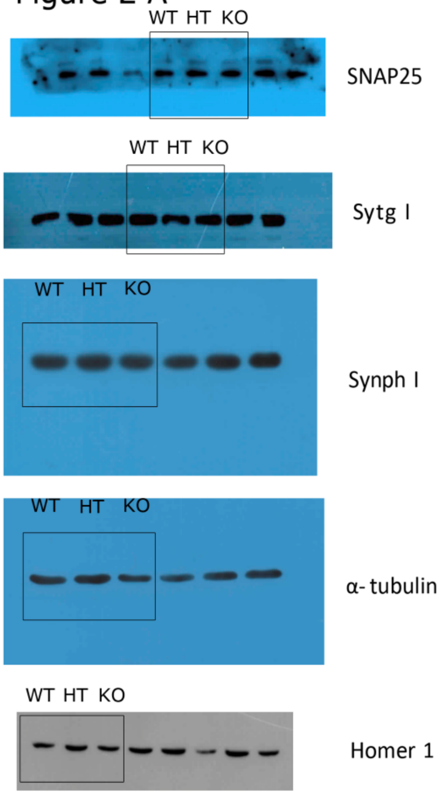

Figure 2 B

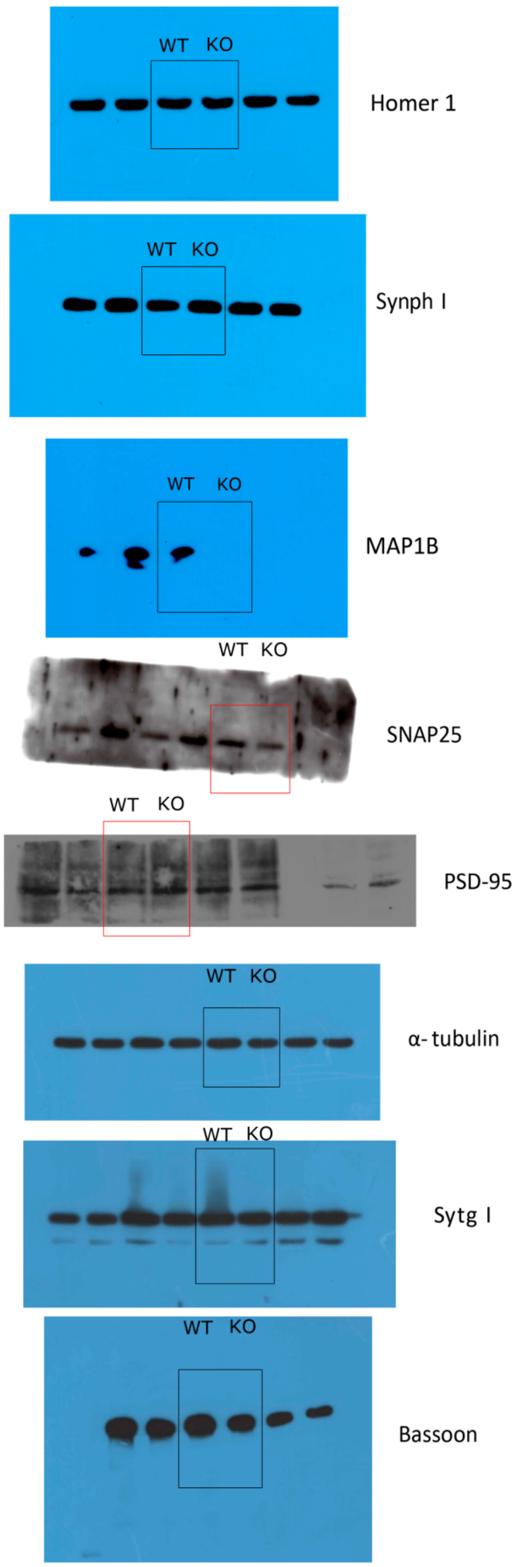

Supplemental Figure 1. FM4-64 dye is not photobleached during image acquisition. FM4-64 normalized fluorescence over time graph from loaded neurons that were not exposed to KCl-induced depolarization or to any change in the medium. During the time of recording (200 sec), no decrease in the fluorescence intensity was detected, indicating that the FM4-64 probe does not suffer photobleaching during the time course analyzed. Graph represents mean ± SEM from 8 different WT neurons.

Supplemental Figure 2. shRNA knockdown efficiently reduces MAP1B protein levels. (A) Mature WT control (GFP-negative) and MAP1B shRNA (GFP-positive) neurons, where white channel correspond to MAP1B, green to GFP and red to β3-tubulin. Yellow strips indicate the proximal-axon transversal area where MAP1B fluorescence profile was analyzed in control and MAP1B shRNA neurons. A red arrowhead indicates the cell body of a MAP1B knockdown neuron. Scale bar 25 μm. **(B)** Fluorescence intensity profile for MAP1B normalized to β3-tubulin signals, show that MAP1B shRNA neuron present a decrease of a 67.37% in the area under the curve when compared to the control neuron. **(C)** Knockdown in neuroblastoma N2a cell line. Cells transfected with MAP1B shRNA show a decrease in the MAP1B/tubulin levels compared to control, t-test $p<0.01$ $n=3$ for each condition.

Supplemental Figure 3. MAP1B-deficient axons establish fewer synapses than control axons. Representative images of mature WT control (GFP-negative) and MAP1B shRNA (GFP-positive) neurons. **(A)** A single WT dendrite is contacted by both WT and MAP1B shRNA axons. White arrows indicate synapses between a presynaptic MAP1B shRNA axon (GFP-positive) and a WT dendrite. Yellow arrowheads indicate synapses between WT presynaptic axon and a WT dendrite. Scale bar 10 μm. **(B)** WT dendrites are innervated by WT and MAP1B shRNA axons. Scale bar 20 μm, inset scale bar 10 μm

Supplemental Figure 4. Morphological parameters of SV. (A) Quantification of the SV diameter in each type of synapse. For WT neurons, asymmetrical synapses are 43.74 ± 0.72 nm, and symmetrical synapses are 42.49 ± 0.68 nm t-test $p<0.05$. For MAP1B KO neurons, asymmetrical synapses are 44.30 ± 0.67 nm, and symmetrical synapses are 42.60 ± 0.70 nm, one–way ANOVA with Bonferroni post-test, n.s. **(B)** Quantification of the SV ellipticity in each type of synapse in neurons of both genotypes. In the equation, x and y represent perpendicular axis traced over the diameter of a SV. The contour of a SV with the average value for ellipticity is drawn. One–way ANOVA with Bonferroni post-test, n.s.

Western Blot raw data. Western blot raw data for available gels. Films were scanned in order to include the cut membranes that were incubated with the respective antibody. Representative cropped sections are highlighted for each blot.
